# APPL Proteins FRET at the BAR: Direct Observation of APPL1 and APPL2 BAR Domain-Mediated Interactions on Cell Membranes Using FRET Microscopy

**DOI:** 10.1371/journal.pone.0012471

**Published:** 2010-08-30

**Authors:** Heidi J. Chial, Peter Lenart, Yong Q. Chen

**Affiliations:** 1 Department of Cancer Biology, Wake Forest University School of Medicine, Winston-Salem, North Carolina, United States of America; 2 Cell Biology and Biophysics Programme, European Molecular Biology Laboratory (EMBL), Heidelberg, Germany; University of Birmingham, United Kingdom

## Abstract

**Background:**

Human APPL1 and APPL2 are homologous RAB5 effectors whose binding partners include a diverse set of transmembrane receptors, signaling proteins, and phosphoinositides. APPL proteins associate dynamically with endosomal membranes and are proposed to function in endosome-mediated signaling pathways linking the cell surface to the cell nucleus. APPL proteins contain an N-terminal Bin/Amphiphysin/Rvs (BAR) domain, a central pleckstrin homology (PH) domain, and a C-terminal phosphotyrosine binding (PTB) domain. Previous structural and biochemical studies have shown that the APPL BAR domains mediate homotypic and heterotypic APPL-APPL interactions and that the APPL1 BAR domain forms crescent-shaped dimers. Although previous studies have shown that APPL minimal BAR domains associate with curved cell membranes, direct interaction between APPL BAR domains on cell membranes *in vivo* has not been reported.

**Methodology:**

Herein, we used a laser-scanning confocal microscope equipped with a spectral detector to carry out fluorescence resonance energy transfer (FRET) experiments with cyan fluorescent protein/yellow fluorescent protein (CFP/YFP) FRET donor/acceptor pairs to examine interactions between APPL minimal BAR domains at the subcellular level. This comprehensive approach enabled us to evaluate FRET levels in a single cell using three methods: sensitized emission, standard acceptor photobleaching, and sequential acceptor photobleaching. We also analyzed emission spectra to address an outstanding controversy regarding the use of CFP donor/YFP acceptor pairs in FRET acceptor photobleaching experiments, based on reports that photobleaching of YFP converts it into a CFP-like species.

**Conclusions:**

All three methods consistently showed significant FRET between APPL minimal BAR domain FRET pairs, indicating that they interact directly in a homotypic (i.e., APPL1-APPL1 and APPL2-APPL2) and heterotypic (i.e., APPL1-APPL2) manner on curved cell membranes. Furthermore, the results of our experiments did not show photoconversion of YFP into a CFP-like species following photobleaching, supporting the use of CFP donor/YFP acceptor FRET pairs in acceptor photobleaching studies.

## Introduction

Human APPL1 and APPL2 proteins are RAB5 effectors that contain an N-terminal Bin/Amphiphysin/Rvs (BAR) domain, a central pleckstrin homology (PH) domain, and a C-terminal phosphotyrosine binding (PTB) domain. The APPL proteins collectively interact with a diverse repertoire of binding partners: transmembrane receptors (the netrin-1 receptor (DCC [1]), the adiponectin receptors (AdipoR1 and AdipoR2 [2], [3], [4]), the follicle-stimulating hormone (FSH) receptor (FSHR [5], [6]), and the nerve growth factor (NGF) receptor (TrkA [7], [8])), signaling proteins (AKT proteins [9], [10] and GIPC1 [7], [8]), small GTPases (RAB5 [11] and RAB22 [12]), components of the nucleosome remodeling and histone deacetylation complex NuRD (MTA2, RBBP7, HDAC1, and HDAC2 [11], [13]), RUVBL2 [14], LKB1 [15], [16], enzymes involved in phosphoinositide metabolism (PI3K [9], OCRL [17], [18], and INPP5B [17]), and phosphoinositides [19], [20]. Furthermore, the APPL proteins form homooligomers (APPL1-APPL1 and APPL2-APPL2) [20] and heterooligomers (APPL1-APPL2) [6], [20]. APPL proteins associate dynamically with endosomal membranes [20], and are proposed to function in an endosome-mediated signaling pathway bridging receptor activation at the cell surface with downstream nuclear signaling events [11].

The crystal structures of the APPL1 BAR, PH, BAR-PH, and PTB domains have been solved [12], [19]. The APPL1 BAR domain structure is distinct from other BAR domains, which consist of three α-helices and associate in an anti-parallel manner with a second BAR domain to form a crescent-shaped dimer. In contrast, the APPL1 BAR domain monomer contains a fourth α-helix that extends away from the first three α-helices and contributes to an extended dimer interface consisting of two bundles of four α-helices; the fourth α-helix is located on the convex face of the BAR domain dimer and does not contribute to the structure of the concave inner face [12], [19]. The APPL minimal BAR domains, which lack the fourth α-helix, are necessary and sufficient for mediating all homotypic and heterotypic APPL-APPL interactions [20]. APPL1 and APPL2 minimal BAR domains associate with curved cell membranes when overexpressed as YFP fusion proteins [20]. Although BAR domains form dimers and associate with curved cell membranes, direct interaction between any of the known BAR domain monomers on cell membranes *in vivo* has not been described.

Fluorescence resonance energy transfer (FRET) microscopy is a powerful tool for determining direct interactions between two proteins at the subcellular level. Often, one protein is fused to cyan fluorescent protein (CFP) as the FRET donor, and the other protein is fused to yellow fluorescent protein (YFP) as the FRET acceptor. Experiments are then carried out to determine whether the proposed protein-binding partners are close enough (i.e., within 1–10 nm of each other) to permit the transfer of energy from the CFP FRET donor to the YFP FRET acceptor, providing strong evidence for a direct interaction. Different FRET methods can be employed to detect FRET signal. In FRET acceptor photobleaching experiments, researchers detect FRET signal as an increase in CFP FRET donor emission when the YFP FRET acceptor is bleached. In recent years, however, the use of CFP donor/YFP acceptor FRET pairs in acceptor photobleaching studies has been called into question based on reports that photobleaching of YFP converted it into a CFP-like species [21], [22], [23], which could mimic FRET signal. In contrast, studies by other investigators have argued against such an artifact [24], [25].

Herein, we employed a comprehensive confocal microscopy approach for FRET studies in cells co-expressing the APPL1 and APPL2 minimal BAR domains as CFP and YFP fusions, which allowed us to determine FRET values in a single cell using three FRET methods. Taken together, our experiments address two distinct questions: 1) Do APPL1 and APPL2 minimal BAR domains interact directly in a homotypic manner (i.e., APPL1-APPL1 and APPL2-APPL2) and heterotypic manner (i.e., APPL1-APPL2) on cell membranes? 2) Can CFP/YFP FRET pairs be used in FRET acceptor photobleaching experiments?

## Results

### APPL minimal BAR domain FRET pairs

**Figure 1:**
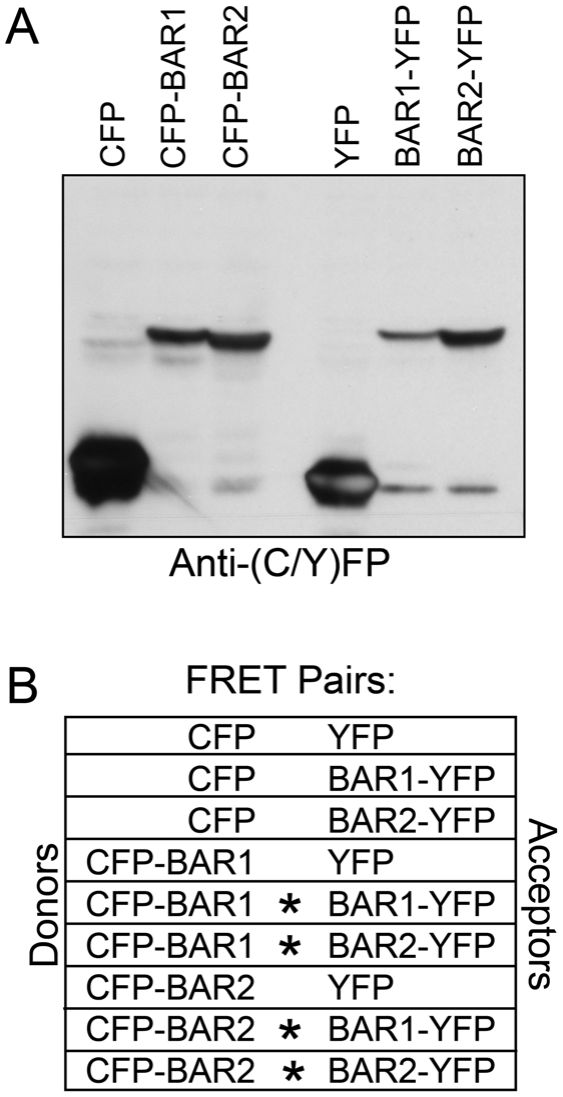
Summary of FRET donors and acceptors. (A) Immunoblot analysis using an antibody that recognizes both CFP and YFP to show appropriate expression and predicted molecular weight for all six FRET donor and acceptor fusion proteins used in these studies. (B) Table showing the nine sets of FRET pairs used in these studies, including five negative control FRET pairs and four experimental FRET pairs (*).

To determine whether APPL minimal BAR domains interact directly on cell membranes, we used a confocal microscopy-based approach for FRET studies in which a single cell was analyzed using three FRET methods resulting in three principally independent data sets. Based on the anti-parallel arrangement of BAR domain dimers, the N terminus of one BAR domain monomer is located in close proximity to the C terminus of the second BAR domain monomer, and vice versa. We used N-terminal CFP fusions of APPL1 and APPL2 minimal BAR domains as FRET donors (CFP-BAR1 and CFP-BAR2, respectively), and we used C-terminal YFP fusions of APPL1 and APPL2 minimal BAR domains as FRET acceptors (BAR1-YFP and BAR2-YFP, respectively); CFP and YFP alone served as negative controls. Therefore, if BAR domain-mediated dimerization occurs, the N-terminal CFP FRET donor of one BAR domain monomer should be located in close proximity to the C-terminal YFP FRET acceptor of the second BAR domain monomer. Immunoblot analysis confirmed that the FRET donor and acceptor proteins were all expressed and of the correct molecular weight (Figure 1A). We co-transfected DLD-1 cells with vectors to express all combinations of FRET donors and acceptors, for a total of nine FRET pairs: four FRET pairs were experimental, and five FRET pairs served as negative controls (Figure 1B). Based on our previous co-localization experiments using a panel of cell membrane compartment markers, the APPL BAR domain-associated membrane structures do not appear to correspond to a known cell membrane system, including early endosomes (RAB5), endoplasmic reticulum (BiP/Grp78), *cis* golgi (GM130), *trans* golgi (TGN38), or caveosomes (caveolin-1) [20]. Therefore, we were unaware of any membrane-associated marker protein that would serve as an appropriate negative control, and we relied on cytosolic CFP and YFP as negative controls in our FRET experiments. Nonetheless, the negative control FRET pairs that included cytosolic CFP and/or cytosolic YFP showed consistently lower FRET values relative to the experimental FRET pairs using all three FRET methods.

### APPL minimal BAR domains interact directly in a homotypic and heterotypic manner on cell membranes

**Figure 2:**
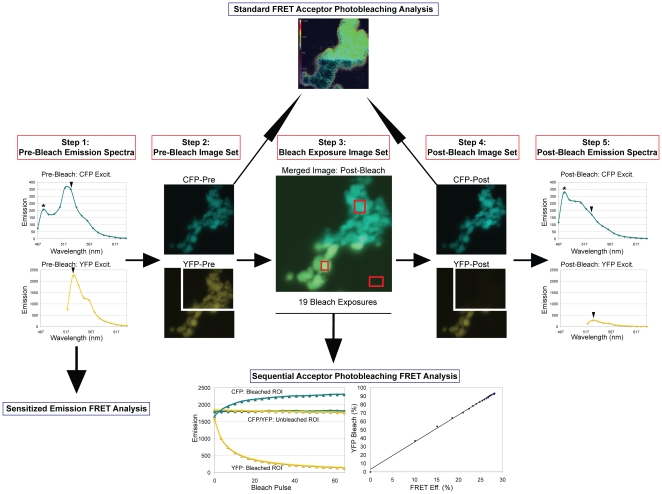
Flow chart of the confocal microscopy approach for FRET studies. The same cell was used in each of the following steps. In Step 1, pre-bleach emission spectra were collected in lambda mode. In Step 2, pre-bleach channel mode images were collected. In Step 3, a boxed cell region was subjected to 19 exposures of acceptor photobleaching, and channel mode images were collected after each of the 19 bleach exposures. Step 4 corresponds to the final post-bleach channel mode image set after the 19 exposures to acceptor photobleaching. In Step 5, post-bleach emission spectra were collected in lambda mode after the 19 exposures to YFP acceptor photobleaching. Three different methods were used to evaluate FRET signal, including sensitized emission (NFRET), standard acceptor photobleaching, and sequential acceptor photobleaching.

**Figure 3:**
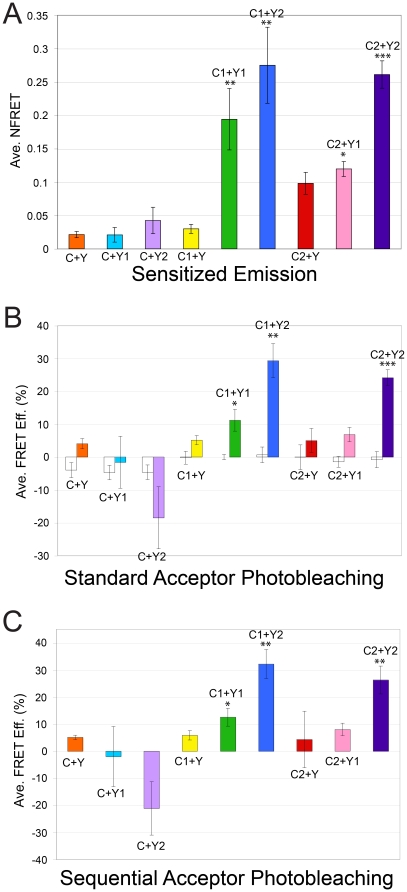
Average FRET values from sensitized emission, standard acceptor photobleaching, and sequential acceptor photobleaching experiments. Data labels: C, C1, C2, Y, Y1, and Y2 correspond to CFP, CFP-BAR1, CFP-BAR2, YFP, BAR1-YFP, and BAR2-YFP, respectively. Statistically significant FRET efficiency values are indicated by *(p-value<0.05), **(p-value≤0.001), and ***(p-value<0.0001). (A) Average NFRET values for sensitized emission studies [26], [27], [28] (Figure S1). (B) Average FRET efficiency values for standard acceptor photobleaching studies [29], [30], [31]. White bars show average FRET efficiency values for unbleached cell regions, and colored bars show average FRET efficiency values for bleached cell regions. (C) Average FRET efficiency values for sequential acceptor photobleaching experiments [32].

**Figure 4:**
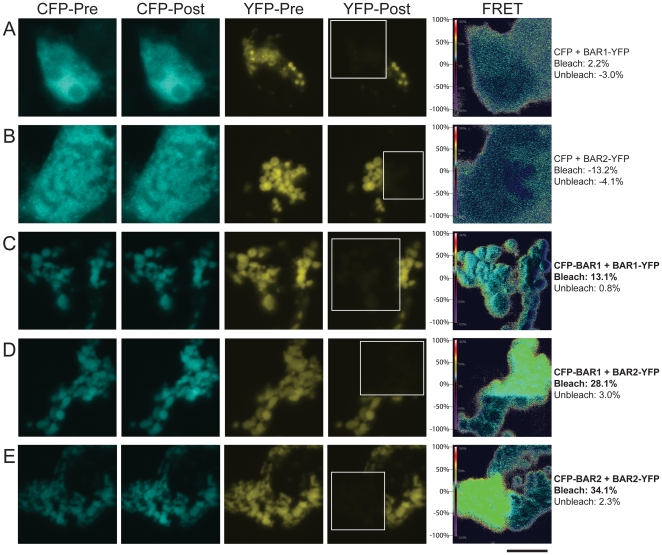
Cell images from standard acceptor photobleaching experiments. Shown are representative cells corresponding to two control FRET pairs: (A) CFP + BAR1-YFP and (B) CFP + BAR2-YFP, and three experimental FRET pairs: (C) CFP-BAR1 + BAR1-YFP, (D) CFP-BAR1 + BAR2-YFP, and (E) CFP-BAR2 + BAR2-YFP analyzed using the standard acceptor photobleaching method. The white box in the YFP-Post image outlines the bleached cell ROI. Average FRET efficiency (%) values within the bleached and unbleached cell regions are shown to the right of each image series. The FRET scale bar (left side of FRET image) shows the corresponding pseudo-colors for FRET efficiency values ranging from −100% to 100%. Bar, 10 µm.

**Figure 5:**
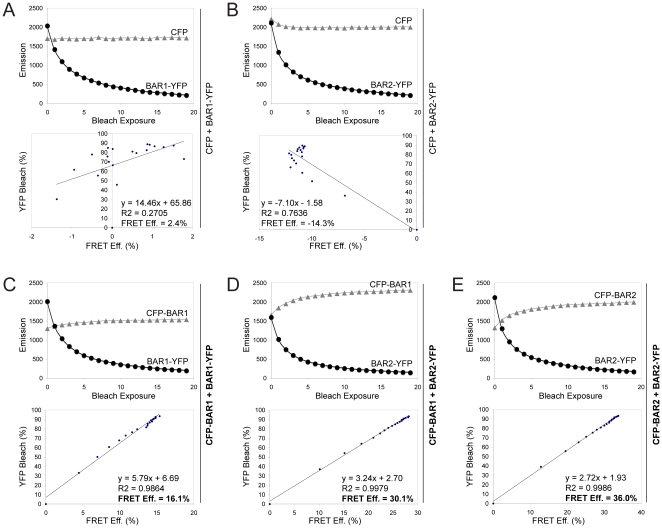
Sequential acceptor photobleaching data. Shown are sequential acceptor photobleaching FRET data from representative cells corresponding to two control FRET pairs: (A) CFP + BAR1-YFP and (B) CFP + BAR2-YFP, and three experimental FRET pairs: (C) CFP-BAR1 + BAR1-YFP, (D) CFP-BAR1 + BAR2-YFP, and (E) CFP-BAR2 + BAR2-YFP. Upper graphs show values for CFP signal (grey triangle) and YFP signal (black circles) within the bleached region of each cell before bleaching and after each of the 19 bleach exposures; these values were used to calculate the FRET efficiency (%) values and the percent decrease in YFP signal after each bleach exposure. The percent decrease in YFP signal and corresponding FRET efficiency values for each of the 20 image sets were plotted and subjected to linear regression analysis to generate equations used to calculate FRET efficiency values when YFP is 100% bleached (lower graph).

**Figure 6:**
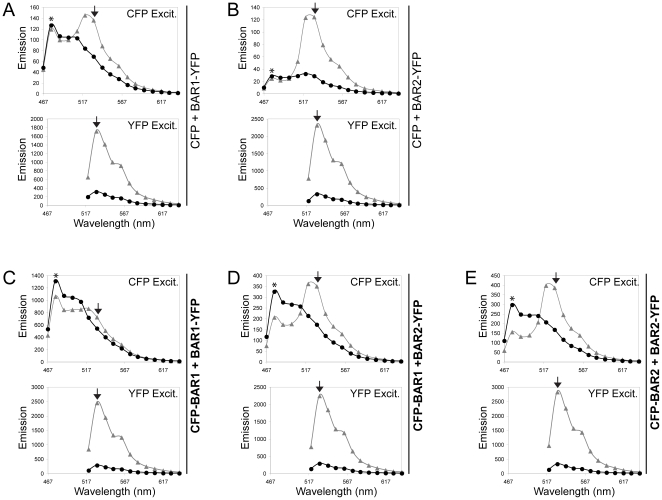
Emission spectra data for FRET pairs. Shown are emission spectra data from representative cells co-expressing control FRET pairs: (A) CFP + BAR1-YFP and (B) CFP + BAR2-YFP, or three experimental FRET pairs: (C) CFP-BAR1 + BAR1-YFP, (D) CFP-BAR1 + BAR2-YFP, and (E) CFP-BAR2 + BAR2-YFP using CFP excitation (CFP Excit., 458 nm) and YFP excitation (YFP Excit., 514 nm). Pre-bleach and post-bleach emission spectra for the same ROI are indicated by grey triangles and black circles, respectively. Peak CFP emission (477 nm) is indicated by an asterisk (*), and peak YFP emission (531 nm) is indicated by the black arrowhead.

As described in the Materials and Methods and summarized in Figure 2, we used a comprehensive multi-step confocal microscopy approach for FRET data collection that allowed us to analyze the same cell using the following three FRET methods: sensitized emission (Figure 3A; Figure S1) [26], [27], [28], standard acceptor photobleaching (Figures 3B & 4) [29], [30], [31], and sequential acceptor photobleaching (Figures 3C & 5) [32]. We also show a comparison of pre-bleach to post-bleach emission spectra (Figure 6). Table S1 shows FRET values for individual cells using the three quantitative FRET methods. We also show average FRET values and standard deviations for each of the nine FRET pairs (Figure 3A, B, & C; Table S2), and we show representative data from the same individual cells for five of the FRET pairs (Figures 4, 5, & 6). Because data from the same representative cells are shown for each of the FRET methods used in Figures 4, 5, and 6, the relative FRET values and trends can be compared directly.

We observed statistically significant FRET values for the CFP-BAR1+BAR1-YFP, CFP-BAR2+BAR2-YFP, and CFP-BAR1+BAR2-YFP FRET pairs relative to negative controls (Figure 3A, B, & C; Table S2) using sensitized emission, standard acceptor photobleaching, and sequential acceptor photobleaching FRET methods. In some cases, negative FRET values were observed in photobleached regions of cells co-expressing the CFP+BAR1-YFP or CFP+BAR2-YFP negative control FRET pairs (Figures 3B, 3C, 4, & 5; Table S1); this is due to the fact that YFP is excited to relatively low levels by the CFP laser (458 nm), and that YFP emission overlaps to a small extent with the bandpass filter used to collect CFP emission (480–520 nm). Therefore, FRET signal must be strong enough to overcome the apparent loss in CFP signal due to YFP photobleaching. We observed statistically significant FRET signal for the fourth experimental FRET pair (CFP-BAR2+BAR1-YFP) only when using the sensitized emission method (Figure 3A; Tables S1 & S2), as acceptor photobleaching underestimates FRET. The sensitized emission FRET calculation takes into account the expression levels of both the FRET donor and acceptor; this method yields relatively higher FRET values for the two experimental FRET pairs that include the BAR1-YFP FRET acceptor, which may be expressed at lower levels than the BAR2-YFP FRET acceptor (Figure 1A).

In summary, we consistently observed significant FRET values for APPL1-APPL1, APPL2-APPL2, and APPL1-APPL2 minimal BAR domain FRET pairs with all three FRET methods in our experiments using a standard laser-scanning microscope equipped with a spectral detector.

### CFP/YFP FRET pairs in acceptor photobleaching experiments

We also compared pre-bleach and post-bleach emission spectra data to determine whether photobleaching of YFP converted it into a CFP-like species, which would be expected to exhibit a CFP-like emission spectrum following photobleaching. When FRET occurs, one should simultaneously observe two shifts in emission spectra with CFP excitation following acceptor photobleaching: (1) a decrease in YFP-associated emission (peak at 531 nm), and (2) an increase in CFP-associated emission (peak at 477 nm). We observed FRET-associated shifts in emission spectra for the CFP-BAR1+BAR1-YFP, CFP-BAR2+BAR2-YFP, and CFP-BAR1+BAR2-YFP FRET pairs, but not for the negative control FRET pairs (Figure 6). Important to the analysis of the emission spectra data is the fact that we used the same detector gain for the pre-bleach and post-bleach data acquisition, thereby allowing us to compare directly the background-subtracted emission spectra without normalization of the data.

**Figure 7:**
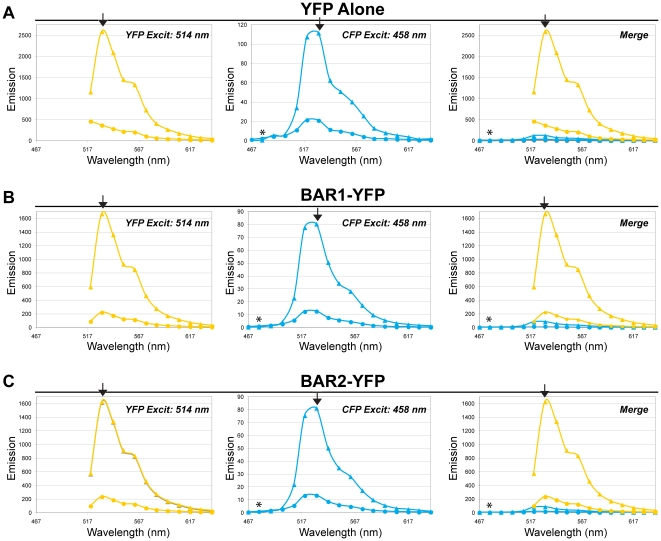
Emission spectra of YFP FRET acceptors do not show photo-conversion of YFP to a CFP-like species after photobleaching. DLD-1 cells were transfected with the YFP FRET acceptors individually, and emission spectra were collected using CFP and YFP excitation wavelengths (458 nm and 514 nm, respectively) before and after 19 bleach exposures (514 nm). Background-subtracted emission values are shown, without any normalization of the data. Shown are representative YFP, CFP, and merged emission spectra for cells expressing (A) YFP alone, (B) BAR1-YFP alone, or (C) BAR2-YFP alone. The left graph shows YFP pre-bleach (triangles) and post-bleach (circles) emission spectra, the center graph shows CFP pre-bleach (triangles) and post-bleach (circles) emission spectra, and the right graph shows the merged data for CFP and YFP emission spectra. Direct excitation of YFP by the CFP laser (excitation: 458 nm; emission: 531 nm) is relatively low, and this signal decreases following YFP photobleaching. Excitation with the CFP laser shows nearly undetectable levels of CFP-like emission signal (excitation: 458 nm; emission: 477 nm) before and after photobleaching. The merged graph shows the relative intensities of emission signals for YFP and CFP excitation; direct comparison is possible because the same detector gain was used to collect all data for a given cell. Peak CFP emission (477 nm) is indicated by an asterisk (*), and peak YFP emission (531 nm) is indicated by the black arrowhead.

In contrast to some reports using CFP/YFP FRET pairs in acceptor photobleaching experiments [21], [22], [23], our comparison of pre-bleach to post-bleach emission spectra using cells co-transfected with negative control FRET pairs failed to uncover evidence for photoconversion of YFP into a CFP-like species. We did not detect photoconversion of YFP into a CFP-like species in cells co-expressing CFP+BAR1-YFP (Figure 6A), in cells co-expressing CFP+BAR2-YFP (Figure 6B), or in cells transfected with any one of the three YFP FRET acceptors individually, including YFP alone (Figure 7A), BAR1-YFP alone (Figure 7B), and BAR2-YFP alone (Figure 7C).

By using the same detector gain to collect all emission spectra from a given cell, we were able to directly compare background-subtracted emission spectra data and avoid complications in interpretation associated with normalization of the data. In these experiments, the CFP FRET donors and YFP FRET acceptors were excited using 458 nm and 514 nm laser settings, respectively, and the peak emissions for CFP and YFP occurred at 477 nm and 531 nm, respectively. Therefore, if photobleaching of YFP converted it into a CFP-like species, one would expect that the photobleached cell region would show an increase in CFP-like emission signal at 477 nm following excitation with the 458 nm CFP laser. In the photobleached region subjected to 19 bleach exposures of the 531 nm YFP laser, cells individually expressing any one of the three YFP FRET acceptors (YFP, BAR1-YFP, or BAR2-YFP) exhibited nearly undetectable levels of CFP emission signal at 477 nm following excitation with the 458 nm CFP laser, and they remained unchanged after any one of the three YFP FRET acceptors were photobleached (Figure 7). As discussed earlier, the YFP FRET acceptors alone exhibited low levels of excitation by the 458 nm laser, which leads to emission signal at 531 nm. As expected, low, but detectable, levels of YFP emission at 531 nm were observed following excitation using the 458 nm laser for each of the YFP FRET acceptors alone before photobleaching; this YFP emission signal at 531 nm decreased significantly when YFP was photobleached.

Taken together, the results of our experiments did not show photoconversion of YFP into a CFP-like species following acceptor photobleaching. These findings support the use of CFP donor/YFP acceptor FRET pairs in acceptor photobleaching experiments.

## Discussion

A previous study demonstrated that endophilin-A1 N-BAR domains dimerize when bound to liposomes *in vitro* [33], and FRET-based approaches have been employed to examine endophilin-A1 N-BAR domain-mediated membrane insertion [34] and membrane fusion [33] events *in vitro*. However, direct interaction between BAR domain monomers on cell membranes *in vivo* has not been reported, and FRET microscopy has not been used to examine BAR domain-mediated dimerization. The FRET studies presented herein provide the first evidence that APPL1 and APPL2 minimal BAR domain monomers interact directly in a homotypic and heterotypic manner on intracellular membranes. All three of the FRET methods employed herein consistently showed significant FRET between APPL1 and APPL2 minimal BAR domain FRET pairs, indicating that they interact directly in a homotypic (i.e., APPL1-APPL1 and APPL2-APPL2) and heterotypic (i.e., APPL1-APPL2) manner on curved cell membranes. Based on our findings and on known BAR domain crystal structures, it is likely that other BAR, N-BAR, and F-BAR domains will exhibit membrane-associated dimerization *in vivo*.

A limitation of the current study is the use of soluble proteins (i.e., CFP and YFP), rather than membrane-targeted proteins, in negative control FRET pairs. As discussed, our previous study showed that the APPL BAR domain-associated cell membrane compartment appears distinct from known membrane systems (i.e., early endosomes, ER, *cis* golgi, *trans* golgi, and caveosomes), and we were unable to include an appropriate membrane-targeted control protein in the current study. Thus, the FRET data from the current study alone are somewhat limited in their demonstration of direct interaction between APPL BAR domains. However, when taken together with previous biochemical experiments (e.g., yeast two-hybrid, co-immunoprecipitation, crystal structures) the FRET data herein provide strong evidence for direct interaction between APPL BAR domains on curved cell membranes. Another limitation of the current study is the dependence of the FRET methods employed on the FRET acceptor∶donor ratio and the fact that the APPL BAR domain FRET donor-acceptor interactions are competing with APPL BAR domain FRET donor-donor and acceptor-acceptor interactions, which would not yield FRET signal. Although significant FRET signal was detected for most of the APPL BAR domain experimental FRET pairs, the absence of significant FRET signal does not necessarily indicate a lack of interaction, as such a lack of significant FRET signal may be due to an unfavorable APPL BAR domain FRET acceptor∶donor ratio as well as a potential preference for APPL BAR domain FRET donor-donor and/or acceptor-acceptor interactions over APPL BAR domain FRET donor-acceptor interactions.

Collectively, our data provide support for the use of CFP/YFP FRET pairs in acceptor photobleaching experiments, and under our experimental conditions, we did not observe that photobleaching of YFP converts it into a CFP-like species [21], [22], [23]. Emission spectra data provide the most accurate insights into the populations of fluorescent species present. By using the same detector gain to collect emission spectra data following CFP and YFP excitation of a given cell, we were able to directly compare background subtracted emission spectra from each cell; this approach eliminated complications associated with normalization of emission spectra data and failed to uncover evidence for the generation of a CFP-like species after YFP FRET acceptors were photobleached. Furthermore, our experiments did not provide evidence for photoconversion of YFP into a CFP-like species in our FRET efficiency calculations using channel mode data from cells co-transfected with negative control FRET pairs. Instead, our channel mode FRET studies required FRET signal to be high enough to overcome an apparent decrease in CFP signal following YFP photobleaching due to cross-talk between CFP and YFP: the CFP laser directly excited YFP, and YFP emission overlapped slightly with the bandwidth filter used to collect CFP emission.

Herein, we used a combination of three different quantitative FRET methods (i.e., sensitized emission, standard acceptor photobleaching, and sequential acceptor photobleaching) to analyze APPL BAR domain interactions. Whereas the data derived from standard acceptor photobleaching and sequential acceptor photobleaching data are overlapping to some extent, they are distinct from data derived from the sensitized emission analysis. Sensitized emission is useful for measuring FRET signal in both fixed and live cell imaging experiments [35], especially when FRET signals are high. Bleed-through can be a source of error in sensitized emission experiments, but appropriate controls can be used to subtract signal due to spectral bleed-through. Standard acceptor photobleaching is one of the more accurate FRET measures because the cells under study serve as their own controls: acceptor photobleaching FRET signal is based solely on changes in CFP FRET donor signal. However, accurate standard acceptor photobleaching FRET measurements require that the FRET donor is not bleached appreciably, that the FRET acceptor is bleached significantly, and that similar concentrations of FRET donors and acceptors are expressed (preferably a donor-to-acceptor ratio between 0.1 to 10) [36]. Sequential acceptor photobleaching is useful when the FRET acceptor is not completely photobleached, as it permits extrapolation to FRET values corresponding to complete (100%) acceptor photobleaching. However, acceptor photobleaching experiments are not very useful for live cell imaging experiments due to the bleach time required and the potential influx of FRET donors/acceptors into the bleached cell region.

The results of our experiments show that sensitized emission analysis can detect even small FRET signals (i.e., CFP-BAR2+BAR1-YFP) not detected with acceptor photobleaching methods. However, sensitized emission is also more prone to errors and can potentially show false positive FRET values for some negative controls (i.e., CFP-BAR2+YFP); this is likely due to the fact that sensitized emission values must be corrected for cross-talk that introduces measurement errors, such as CFP and YFP bleed-through, which contribute to 37% and 4.6% of the FRET signal, respectively. In contrast, false positive FRET values are unlikely using acceptor photobleaching methods. However, due to YFP cross-talk, the acceptor photobleaching method slightly underestimates FRET signal, which may mask small FRET values (i.e., CFP-BAR2+BAR1-YFP). Taken together, the results of this study provide support for the use of a combination of complementary FRET methods.

The approach used herein allowed us to use the same confocal microscope to collect three different types of FRET data in a series of steps using the same cell. Although we employed a comprehensive approach and distinct FRET methods to analyze APPL BAR domain-mediated interactions, we were unable to use fluorescence lifetime imaging microscopy (FLIM) in the current study. FLIM permits accurate FRET measurements based solely on changes in donor emission fluorescence lifetime due to the transfer of energy to the FRET acceptor and is a highly regarded and rigorous FRET method [37]. However, FLIM imaging systems are very complex and sensitive to environmental factors beyond FRET signal itself. Furthermore, CFP has a complex lifetime, which makes it difficult to use for FLIM measurements.

Our analyses suggest that data from experimental FRET pairs should be compared carefully to data from all appropriate negative control FRET pairs in order to determine whether FRET values are significant. Furthermore, the required correction methods and controls also depend on the relative expression levels of the FRET donor and acceptor. Finally, using more than one method to determine FRET values for the same cell provides independent verification of the data. This comprehensive confocal microscopy approach to FRET analysis may be broadly useful for the characterization of direct protein-protein interactions in fixed cells.

In addition to their ability to undergo BAR domain-mediated dimerization and membrane targeting, APPL proteins exhibit PH and PTB domain-mediated phosphoinositide binding [19], [20] and membrane targeting [20]. Dynamic associations between APPL proteins and cell membranes are likely to be coordinately regulated by BAR domain-mediated dimerization, phosphoinositide binding, and interactions with protein binding partners, including transmembrane receptors, signaling proteins, and GTP-bound RAB5. The APPL1 BAR and PH domains are required for interaction with GTP-RAB5 [11]. Analysis of the APPL1 BAR-PH domain crystal structure together with *in vitro* binding studies suggests that APPL1 BAR-PH homodimers form heterotypic RAB5 binding platforms in which the BAR domain of one monomer and the PH domain of a second monomer interact with GTP-RAB5 on each end of the curved BAR-PH dimer [12]. Although GTP-RAB5 interacts with both APPL1 and APPL2 [11], direct interaction between GTP-RAB5 and APPL1 homodimers, APPL2 homodimers, or APPL1-APPL2 heterodimers on cell membranes has not been demonstrated. However, overexpression of APPL1-YFP or APPL2-YFP leads to the recruitment of endogenous RAB5 to enlarged APPL-associated cytosolic membrane structures [20].

Taken together, it is likely that BAR domain-mediated dimerization contributes to the dynamic association between full-length APPL proteins and cell membranes [20], their ability to interact with GTP-bound RAB5 on endosomal membranes [11], and their proposed role in endosome-mediated signal transduction [11]. In summary, our study employed a comprehensive confocal microscopy FRET approach and provides the first direct evidence for BAR domain-mediated homodimerization and heterodimerization on cell membranes *in vivo* by the APPL1 and APPL2 minimal BAR domains.

## Materials and Methods

### FRET donors and acceptors

Clones for the expression of APPL1 and APPL2 minimal BAR domains (APPL1: residues 18–226, and APPL2: residues 18–225) in which YFP was fused to the C terminus of the APPL1 and APPL2 BAR domains were published previously [20]. Clones for the expression of the same residues of the APPL1 and APPL2 BAR domains as CFP fusion proteins in which CFP was fused to the N terminus of the APPL1 and APPL2 BAR domains were generated using the pdECFP vector [38]. Clones for the expression of CFP alone or YFP alone were also published previously [20]. Immunoblot analysis confirmed that the FRET donor and acceptor proteins were all expressed and of the correct molecular weight (Figure 1A).

### Cell culture and transfection conditions

Cells from the human epithelial colorectal cancer cell line DLD-1 (ATCC Number CCL-221) were grown on coverslips and were co-transfected with nine different FRET pairs (Figure 1B); the DLD-1 cells were also transfected individually with each FRET donor or acceptor alone as controls. Lipofectamine 2000 (Invitrogen Corporation, Carlsbad, CA) was used according to the manufacturer's instructions for transfections with 0.8 µg total maxiprep DNA in each well of a 24-well plate; when cells were co-transfected with two different vectors, 0.4 µg of each vector was used. At 24 hours post-transfection, the cells were rinsed with PBS and fixed for 15 min with 2% formaldehyde, followed by PBS washes. The coverslips were mounted using Prolong Gold antifade reagent (Invitrogen Corporation, Carlsbad, CA). All of the data shown herein were derived from the same transfection experiment done in duplicate.

### Confocal microscopy for FRET studies

We used a Zeiss LSM 510 META microscope (Carl Zeiss Inc., Thornwood, NY) equipped with a Zeiss Plan-Apochromat 63×/1.4 NA oil immersion DIC lens, and an argon laser for CFP (458 nm, 0.5% laser power) and YFP excitation (514 nm, 0.4% laser power) with a scan zoom of 6.0, four line averaging, an open pinhole, and an image size of 24.4 µm×24.4 µm. We used a completely open pinhole in order to maximize the detected signal, although this resulted in decreased confocality. Following excitation, channel mode images were collected using band pass filters for CFP emission (BP 480–520 IR) or YFP emission (BP 535–590 IR); channel mode detector gain was set so that neither CFP nor YFP images contained saturated pixels prior to bleaching, but it could differ for CFP and YFP excitation (as we had to use two different photomultiplier tube [PMT] detectors for the two channels). For some of the strong FRET pairs, saturated pixels appeared within the CFP image after photobleaching due to increased CFP emission; in these cases, only cell regions without saturated pixels were used to calculate FRET values.

The META detector is a polychromatic multi-channel detector that allows separation of emission signal into 32 channels with wavelengths ranging from UV to near infrared at approximately 10 nm intervals. We used the same laser settings for lambda mode excitation of CFP and YFP and for channel mode excitation of CFP and YFP (458 nm, 0.5% laser power and 514 nm, 0.4% laser power, respectively); we also used the same detector gain setting for the collection of CFP and YFP emission spectra for each cell before and after bleaching. In all cases, the lambda mode detector gain was set so that no saturated pixels were present in the lambda stack of images for either CFP or YFP excitation prior to photobleaching, and the same detector gain was used for both CFP and YFP excitation of each cell. For CFP and YFP excitation, we analyzed emission from 467–638 nm and 520–638 nm, respectively.

We used the Zeiss bleach control to select a boxed region of interest (ROI) within each cell for acceptor photobleaching: the ROI was subjected to 19 exposures to the YFP laser (514 nm, 100% laser power) for one-second intervals.

### FRET data collection

In these studies, we analyzed individual cells using confocal microscopy with sequential acceptor photobleaching within a selected cell region. This approach allowed us to evaluate FRET signals in the same cell using three methods for FRET analysis. After identifying a co-transfected cell, we selected a boxed ROI within the cell for acceptor photobleaching. We set the channel mode detector gain so that neither CFP nor YFP images contained saturated pixels prior to bleaching. For data acquired from a given cell in channel mode, we collected pre-bleach and post-bleach images using the same detector gain, but the same detector gain was not necessarily used for CFP and YFP excitation. We also set the lambda mode detector gain so that no saturated pixels were present in the lambda stack of images for either CFP or YFP excitation prior to photobleaching. For data collected from a given cell in lambda mode, we collected the pre-bleach and post-bleach emission spectra using the same detector gain, and we also used the same detector gain for both CFP and YFP excitation.

We used the same cell in each of the following steps (shown in Figure 2). In Step 1, we first collected pre-bleach emission spectra of the entire cell using CFP and YFP excitation wavelengths (458 nm and 514 nm, respectively) in lambda mode. In Step 2, we switched to channel mode and collected a pre-bleach channel mode image set using CFP excitation/emission and YFP excitation/emission settings. In Step 3, we subjected the selected cell region to 19 exposures of acceptor photobleaching (YFP excitation at 100% laser power for one-second intervals) and collected CFP and YFP channel mode images of the entire cell after each of the 19 bleach exposures. After 19 bleach exposures, the FRET acceptor was nearly completely bleached within the selected cell region. The final channel mode image set captured after the last bleach exposure corresponded to the post-bleach channel mode image set (Step 4). In Step 5, we then returned to lambda mode and collected post-bleach emission spectra of the entire cell using CFP and YFP excitation wavelengths.

For each of the nine FRET pairs, we collected complete FRET data sets for five individual cells (with the exception of the APPL2-CFP+APPL2-YFP FRET pair for which we collected data sets from seven individual cells). We then analyzed the data using three FRET methods for each individual cell, including sensitized emission [26], [27], [28], standard acceptor photobleaching [29], [30], [31], and sequential acceptor photobleaching [32]. The data sets acquired by the three different methods can principally be considered independent, because we used data collected using different detectors, and we did not select identical cell regions to extract donor/acceptor signal or emission spectra data for any of the quantitative methods.

### Sensitized emission FRET analysis

Sensitized emission relies on detection of emission of acceptor fluorescence upon excitation of the donor fluorophore [26], [27], [28]. In theory, all acceptor (YFP) emission results from energy transfer from the FRET donor (CFP) excitation. However, donor emission (CFP, 467–638 nm) overlaps with acceptor emission (YFP, 520–638 nm), and the CFP laser can also directly excite YFP leading to YFP emission that is not due to FRET [26], [27]. Additionally, the relative expression levels of the FRET donor and acceptor within a given cell must be taken into account, since they are not necessarily expressed in a 1∶1 manner [27], [28].

To evaluate cross-talk between CFP and YFP, we used emission spectra data from cells expressing each FRET donor or acceptor alone to calculate spectral bleed-through constants using values at the peak emission wavelengths for CFP and YFP (477 nm and 531 nm, respectively) [26], [27], [28]. We used the same detector gain when collecting both CFP and YFP emission spectra from a given cell, and all of the data used in our calculations were derived from background-subtracted emission values without normalization of the data. We calculated CFP bleed-through constants (CFP_BT_) using the emission spectra data for CFP excitation of the FRET donor-alone transfected cells: signal at the peak YFP emission wavelength (531 nm) was divided by signal at the peak CFP emission wavelength (477 nm) (Figure S1A). In our experiments, the average CFP_BT_ value was 0.37+/−0.009. We calculated YFP bleed-through constants (YFP_BT_) using the emission spectra data for both CFP and YFP excitation using the FRET acceptor-alone transfected cells: signal at the peak YFP emission wavelength (531 nm) using CFP excitation was divided by signal at 531 nm using YFP excitation (Figure S1B). In our experiments, the average YFP_BT_ value was 0.046+/−0.004.

To evaluate cross-talk between CFP and YFP, we used the average CFP and YFP bleed-through constants in our calculations to determine normalized FRET (NFRET) values [28] for cells co-transfected with each of the nine FRET pairs. Using our pre-bleach emission spectra data, we determined the emission signal at 531 nm with CFP excitation (FRET), emission signal at 477 nm with CFP excitation (CFP), and emission signal at 531 nm with YFP excitation (YFP) for each cell (Figure S1C). To account for cross-talk between CFP and YFP, we used the following formula:
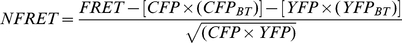
to determine the NFRET value (Figure S1D). Tables S1 and S2 show the sensitized emission values for each individual cell and the average sensitized emission values for each FRET pair, respectively.

### Standard acceptor photobleaching FRET experiments

We used the pre-bleach channel mode image and the final post-bleach channel mode image (after 19 bleach exposures) for standard acceptor photobleaching FRET studies. We used Zeiss imaging software to concatenate the pre-bleach and post-bleach image sets and generated pseudo-colored images showing FRET efficiency values throughout each cell; all images were background-subtracted. FRET efficiency values were calculated using the following equation:
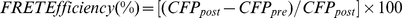
where CFP_pre_ corresponds to the background corrected CFP signal before bleaching, and CFP_post_ corresponds to the final CFP signal after 19 bleach exposures within the same ROI [29], [30], [31]. Within each cell, we selected five boxed ROIs in the bleached cell region, and five boxed ROIs in the unbleached cell region. We then used the Zeiss FRET software to calculate FRET efficiency values for each ROI and determined average FRET efficiency values within the bleached and unbleached ROIs. In many cases, the unbleached ROI shows a small, negative FRET value. This is due to a small decrease in CFP signal caused by exposure to the laser during the acquisition of 20 channel mode images (one pre-bleach, followed by 19 post-bleach exposure images). If CFP_post_ is lower than CFP_pre_, the standard acceptor photobleaching equation will yield a negative FRET efficiency value.

Under the experimental conditions used herein, we observed cross-talk between CFP and YFP: the 458 nm laser setting used to excite CFP was capable of direct YFP excitation, and the BP480–520 filter set used to detect CFP emission in channel mode also detected YFP emission. However, the BP480–520 filter is appropriate for use in CFP-YFP FRET studies. Our YFP bleed-through constant calculations using emission spectra data show that direct excitation of YFP by the 458 nm laser is relatively low (YFP_BT_ = 0.046+/−0.004 at 531 nm). When YFP is bleached, there will be a slight decrease in apparent CFP signal due to YFP bleed-through and loss of this signal when YFP is photobleached. Therefore, the BP480–520 filter would only present problems if FRET signal were not strong enough to overcome the relatively low decrease in CFP signal due to YFP bleaching and loss of the corresponding YFP bleed-through signal. As a result, this method slightly underestimates FRET. For example, examination of the sequential acceptor photobleaching data for the CFP+BAR2-YFP negative control FRET pair shows a small exponential decrease in CFP signal following photobleaching (Figure 5B); this is due to BAR2-YFP bleed-through and exponential decay of “CFP” signal as YFP is bleached. Therefore, FRET signal in our studies must be high enough to overcome this apparent decrease in donor signal due to loss of YFP bleed-through signal during acceptor photobleaching. Table S1 shows the standard acceptor photobleaching FRET efficiency values in a bleached and unbleached region of each individual cell, and Table S2 shows the average FRET efficiency values for each FRET pair.

### Sequential acceptor photobleaching FRET experiments

The sequential acceptor photobleaching experiments utilized all 20 channel mode images (one pre-bleach image and 19 post-bleach images). The data were collected in series, so could be analyzed as a stack of images in chronological order. We selected an ROI within the bleached cell region, an ROI within the unbleached cell region, and a background ROI. We then used the Zeiss software to obtain values for CFP and YFP signal within each ROI for each of the 20 individual images within the image stack. The CFP and YFP signals within the bleached or unbleached ROIs were background-subtracted. We then plotted the changes in CFP and YFP signal after each bleach exposure. Within the unbleached cell ROIs, no significant changes in CFP or YFP signal were observed; these data were not used in subsequent calculations. Within the bleached cell ROIs, YFP signal always exhibited an exponential decay following the bleach exposures. Changes in CFP signal varied, depending on the FRET pair. In general, when FRET occurs, the exponential decrease in FRET acceptor (YFP) signal within the bleached ROI should be accompanied by a corresponding exponential increase in FRET donor (CFP) signal following the bleach exposures [32].

Within the bleached cell ROI, values for YFP signal before bleaching (YFP_pre_) and after each bleach exposure (YFP_bleach_) were used to calculate the percent decrease in YFP signal after each bleach exposure:
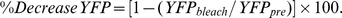
Within the bleached cell region, values for CFP signal before bleaching (CFP_pre_) and after each bleach exposure (CFP_bleach_) were used to calculate the FRET efficiency (%) values after each bleach exposure [29], [30], [31]:
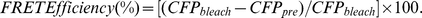
The pre-bleach image has values of 0% for both the decrease in YFP signal and the FRET efficiency. After each bleach exposure, the value for the percent decrease in YFP signal approached 100%. When FRET occurs, a linear relationship should exist between the % decrease in YFP signal and the increase in FRET efficiency (%) after each bleach exposure, and the equation for this line can be used to determine the FRET efficiency (%) value when the acceptor is completely bleached (100% decrease in YFP). For each cell examined, we plotted the % YFP decrease vs. FRET efficiency (%), followed by linear regression analysis [39], [40], and we used the linear equation to determine the corresponding FRET efficiency (%) value when YFP is bleached to completion (100% YFP decrease). Sequential acceptor photobleaching data from representative cells are shown (Figure 5B), and FRET efficiency values for individual cells and average FRET efficiency values for each FRET pair are shown in Tables S1 and S2, respectively.

### Comparison of pre-bleach and post-bleach emission spectra

Emission spectra data for each cell were collected using the same detector gain for CFP excitation pre-bleach, YFP excitation pre-bleach, CFP excitation post-bleach, and YFP excitation post-bleach. The post-bleach emission spectra were collected after the cells had been subjected to 19 bleach exposures. To compare emission spectra data of a given cell, we concatenated the pre-bleach and post-bleach emission spectra data sets for both CFP and YFP excitation. We then selected three ROIs: 1) a bleached cell region, 2) an unbleached cell region, and 3) a background region. Because we used the same detector gain to collect the pre-bleach and post-bleach emission spectra, our data represent actual background-subtracted emission values without any normalization of the data. We only show pre-bleach and post-bleach emission spectra for CFP and YFP excitation of each cell within the bleached cell region, because no significant changes in emission spectra were observed in unbleached cell regions.

### Statistical analyses of FRET values

The statistical significance of the FRET values was determined by Student's t-tests and pair-wise comparisons of the BAR domain-containing FRET pairs to three appropriate negative control FRET pairs to obtain p-values; either equal (Pooled method) or unequal (Satterthwaite method) variances were used, depending on whether the F-test comparing the variances was significant (if non-significant, equal variance test was used; if significant, unequal variance test was used) (Table S2). For example, the CFP-BAR1 + BAR1-YFP FRET pair results were compared to the CFP + YFP, CFP-BAR1 + YFP, and CFP + BAR1-YFP negative control FRET pair results (Table S2). The CFP-BAR1 + BAR2-YFP FRET pair results were compared to the CFP + YFP, CFP-BAR1 + YFP, and CFP + BAR2-YFP negative control FRET pair results (Table S2). The CFP-BAR2 + BAR1-YFP FRET pair results were compared to the CFP + YFP, CFP-BAR2 + YFP, and CFP+BAR1-YFP negative control FRET pair results (Table S2). The CFP-BAR2 + BAR2-YFP FRET pair results were compared to the CFP + YFP, CFP-BAR2 + YFP, and CFP+BAR2-YFP negative control FRET pair results (Table S2). The indicated level of statistical significance for each BAR domain FRET pair is based on the least significant of the three pair-wise comparisons (* for p-values less than 0.05, ** for p-values less than or equal to 0.001, and *** for p-values less than 0.0001).

## Supporting Information

Figure S1 Summary of the sensitized emission FRET protocol using pre-bleach emission spectra data. (A) CFP bleed-through constants (CFP_BT_) were determined for each FRET donor (CFP, CFP-BAR1, and CFP-BAR2) by analyzing emission spectra from cells expressing only the FRET donor [26], [27], [28]. The emission signal at 531 nm [A] was divided by emission signal at 477 nm (B) using CFP excitation; the average CFP_BT_ value was 0.37±0.009. (B) YFP bleed-through constants (YFP_BT_) were determined for each FRET acceptor (YFP, BAR1-YFP, and BAR2-YFP) by analyzing emission spectra from cells expressing only the FRET acceptor. The emission signal at 531 nm using CFP excitation [C] was divided by the emission signal at 531 nm using YFP excitation [D]; the average YFP_BT_ value was 0.046±0.004. (C) Sensitized emission FRET values were calculated using emission spectra from cells co-expressing the FRET donor and acceptor. FRET signal due to direct CFP excitation was determined by multiplying emission signal at 477 nm with CFP excitation [CFP] by CFP_BT_. To evaluate cross-talk between CFP and YFP, FRET signal due to direct YFP excitation by the CFP laser was determined by multiplying the emission signal at 531 nm with YFP excitation [YFP] by YFP_BT_. (D) Normalized FRET signal (NFRET) was determined by subtracting FRET signal due to CFP bleed-through [CFP×CFP_BT_] and YFP bleed-through [YFP×YFP_BT_] from the preliminary FRET value [FRET], which was then divided by the square root of the product of CFP and YFP signal to normalize for differences in expression levels of the FRET donor and acceptor.
(0.58 MB TIF)

Table S1 Summary of FRET values for each individual cell analyzed. (* Indicates representative cells shown in Figures 4, 5, & 6.)
(0.40 MB DOC)

Table S2 Summary of average FRET values, standard deviations, and p-values for the nine FRET pairs.
(0.14 MB DOC)
