# Supplementary figures and images for: APPL Proteins FRET at the BAR: Direct Observation of APPL1 and APPL2 BAR Domain-Mediated Interactions on Cell Membranes Using FRET Microscopy
Source: PLoS One. 2010 Aug 30;5(8):e12471. doi: 10.1371/journal.pone.0012471 (PMC2930004; doi:10.1371/journal.pone.0012471)

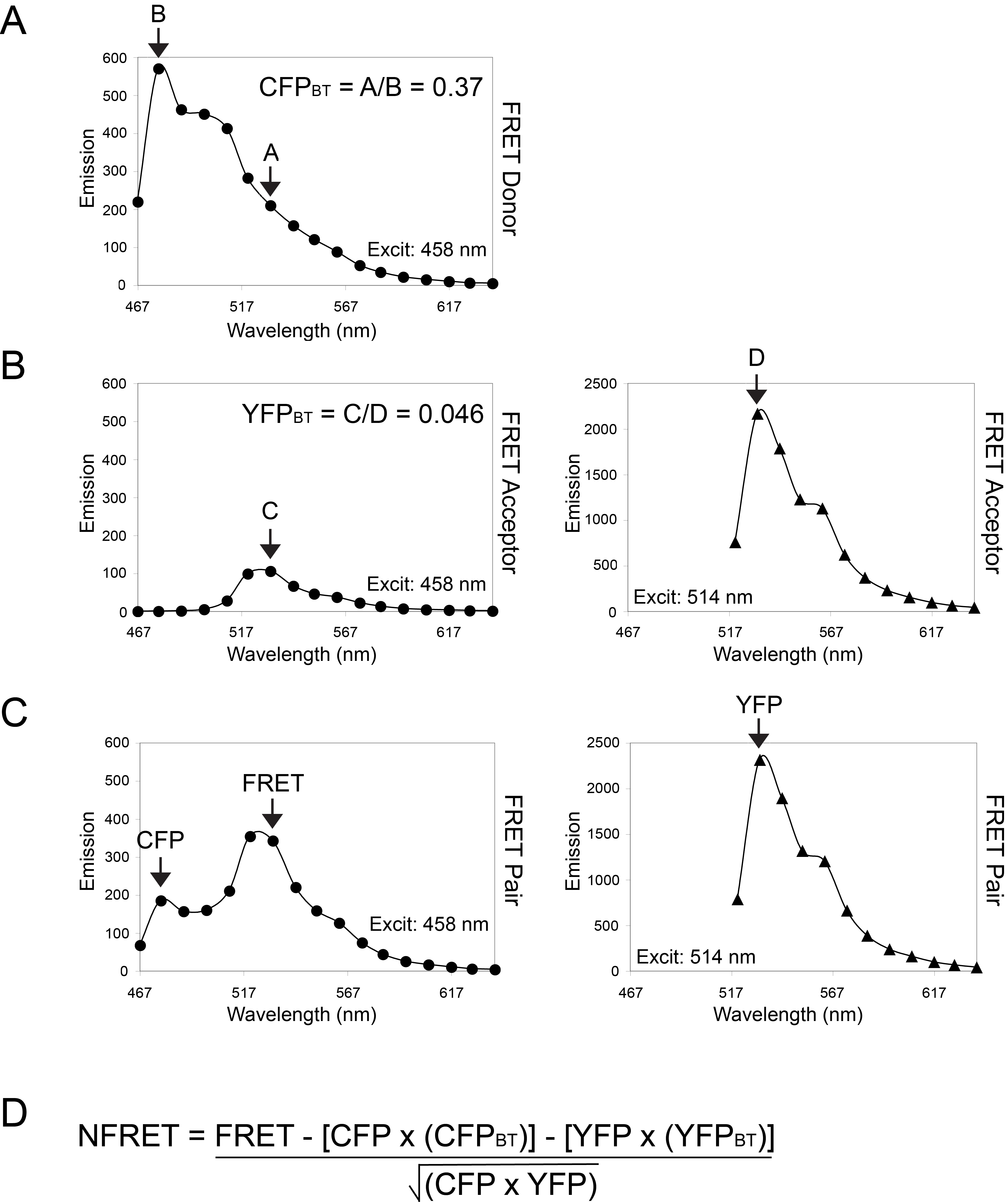

Supplement: Figure S1 — Summary of the sensitized emission FRET protocol using pre-bleach emission spectra data. (A) CFP bleed-through constants (CFPBT) were determined for each FRET donor (CFP, CFP-BAR1, and CFP-BAR2) by analyzing emission spectra from cells expressing only the FRET donor [26], [27], [28]. The emission signal at 531 nm [A] was divided by emission signal at 477 nm (B) using CFP excitation; the average CFPBT value was 0.37±0.009. (B) YFP bleed-through constants (YFPBT) were determined for each FRET acceptor (YFP, BAR1-YFP, and BAR2-YFP) by analyzing emission spectra from cells expressing only the FRET acceptor. The emission signal at 531 nm using CFP excitation [C] was divided by the emission signal at 531 nm using YFP excitation [D]; the average YFPBT value was 0.046±0.004. (C) Sensitized emission FRET values were calculated using emission spectra from cells co-expressing the FRET donor and acceptor. FRET signal due to direct CFP excitation was determined by multiplying emission signal at 477 nm with CFP excitation [CFP] by CFPBT. To evaluate cross-talk between CFP and YFP, FRET signal due to direct YFP excitation by the CFP laser was determined by multiplying the emission signal at 531 nm with YFP excitation [YFP] by YFPBT. (D) Normalized FRET signal (NFRET) was determined by subtracting FRET signal due to CFP bleed-through [CFP×CFPBT] and YFP bleed-through [YFP×YFPBT] from the preliminary FRET value [FRET], which was then divided by the square root of the product of CFP and YFP signal to normalize for differences in expression levels of the FRET donor and acceptor. (0.58 MB TIF) [file pone.0012471.s001.tif]
