# Supplementary material for: APPL Proteins FRET at the BAR: Direct Observation of APPL1 and APPL2 BAR Domain-Mediated Interactions on Cell Membranes Using FRET Microscopy
Source: PLoS One. 2010 Aug 30;5(8):e12471. doi: 10.1371/journal.pone.0012471 (PMC2930004; doi:10.1371/journal.pone.0012471)
Supplement: Table S1 — Summary of FRET values for each individual cell analyzed. (* Indicates representative cells shown in Figures 4, 5, & 6.) (0.40 MB DOC) [file pone.0012471.s002.doc]

**Table S1. Summary of FRET values for each individual cell analyzed. (* Indicates representative cells shown in Figures 4, 5, & 6)**

| **CFP + YFP**  **FRET Pair** | **Sensitized Emission**  **NFRET** | **Standard APB**  **FRET Eff. (%)** | **Standard APB**  **Cell Region** | **Sequential APB**  **FRET Eff. (%)** | **Sequential APB**  **R2 Value** |
| --- | --- | --- | --- | --- | --- |
|  |  |  |  |  |  |
| **Cell 1** | 0.0158 | 5.64 | Bleached | 5.24 | 0.9161 |
|  |  | -4.94 | Unbleached |  |  |
|  |  |  |  |  |  |
| **Cell 2** | 0.0173 | 2.70 | Bleached | 5.24 | 0.4704 |
|  |  | -4.98 | Unbleached |  |  |
|  |  |  |  |  |  |
| **Cell 3** | 0.0234 | 2.14 | Bleached | 3.76 | 0.6409 |
|  |  | -0.17 | Unbleached |  |  |
|  |  |  |  |  |  |
| **Cell 4** | 0.0273 | 4.47 | Bleached | 5.41 | 0.8998 |
|  |  | -3.85 | Unbleached |  |  |
|  |  |  |  |  |  |
| **Cell 5** | 0.0224 | 5.34 | Bleached | 5.96 | 0.5179 |
|  |  | -5.99 | Unbleached |  |  |
|  |  |  |  |  |  |

| **CFP + BAR1-YFP**  **FRET Pair** | **Sensitized Emission**  **NFRET** | **Standard APB**  **FRET Eff. (%)** | **Standard APB**  **Cell Region** | **Sequential APB**  **FRET Eff. (%)** | **Sequential APB**  **R2 Value** |
| --- | --- | --- | --- | --- | --- |
|  |  |  |  |  |  |
| **Cell 1** | 0.0143 | 3.05 | Bleached | 4.03 | 0.9048 |
|  |  | -7.57 | Unbleached |  |  |
|  |  |  |  |  |  |
| **Cell 2** | 0.0305 | -15.61 | Bleached | -21.83 | 0.8471 |
|  |  | -6.17 | Unbleached |  |  |
|  |  |  |  |  |  |
| **Cell 3*** | 0.0338 | 2.16 | Bleached | 2.36 | 0.2705 |
|  |  | -2.95 | Unbleached |  |  |
|  |  |  |  |  |  |
| **Cell 4** | 0.0186 | 2.07 | Bleached | 2.56 | 0.2314 |
|  |  | -4.71 | Unbleached |  |  |
|  |  |  |  |  |  |
| **Cell 5** | 0.0080 | 0.38 | Bleached | 2.86 | 0.4370 |
|  |  | -2.32 | Unbleached |  |  |
|  |  |  |  |  |  |

| **CFP + BAR2-YFP**  **FRET Pair** | **Sensitized Emission**  **NFRET** | **Standard APB**  **FRET Eff. (%)** | **Standard APB**  **Cell Region** | **Sequential APB**  **FRET Eff. (%)** | **Sequential APB**  **R2 Value** |
| --- | --- | --- | --- | --- | --- |
|  |  |  |  |  |  |
| **Cell 1*** | 0.0478 | -13.20 | Bleached | -14.32 | 0.7636 |
|  |  | -4.06 | Unbleached |  |  |
|  |  |  |  |  |  |
| **Cell 2** | 0.0195 | -9.57 | Bleached | -11.14 | 0.7124 |
|  |  | -3.12 | Unbleached |  |  |
|  |  |  |  |  |  |
| **Cell 3** | 0.0723 | -22.78 | Bleached | -25.35 | 0.9120 |
|  |  | -4.17 | Unbleached |  |  |
|  |  |  |  |  |  |
| **Cell 4** | 0.0413 | -33.03 | Bleached | -35.93 | 0.9328 |
|  |  | -8.54 | Unbleached |  |  |
|  |  |  |  |  |  |
| **Cell 5** | 0.0316 | -13.97 | Bleached | -18.86 | 0.7832 |
|  |  | -3.27 | Unbleached |  |  |
|  |  |  |  |  |  |

| **CFP-BAR1 + YFP**  **FRET Pair** | **Sensitized Emission**  **NFRET** | **Standard APB**  **FRET Eff. (%)** | **Standard APB**  **Cell Region** | **Sequential APB**  **FRET Eff. (%)** | **Sequential APB**  **R2 Value** |
| --- | --- | --- | --- | --- | --- |
|  |  |  |  |  |  |
| **Cell 1** | 0.0242 | 3.32 | Bleached | 3.44 | 0.8136 |
|  |  | -3.37 | Unbleached |  |  |
|  |  |  |  |  |  |
| **Cell 2** | 0.0220 | 6.95 | Bleached | 7.98 | 0.9856 |
|  |  | 1.91 | Unbleached |  |  |
|  |  |  |  |  |  |
| **Cell 3** | 0.0307 | 4.96 | Bleached | 7.08 | 0.9737 |
|  |  | 0.004 | Unbleached |  |  |
|  |  |  |  |  |  |
| **Cell 4** | 0.0350 | 4.40 | Bleached | 5.27 | 0.9834 |
|  |  | -0.28 | Unbleached |  |  |
|  |  |  |  |  |  |
| **Cell 5** | 0.0376 | 5.97 | Bleached | 5.63 | 0.9095 |
|  |  | 0.66 | Unbleached |  |  |
|  |  |  |  |  |  |

| **CFP-BAR1 +**  **BAR1-YFP**  **FRET Pair** | **Sensitized Emission**  **NFRET** | **Standard APB**  **FRET Eff. (%)** | **Standard APB**  **Cell Region** | **Sequential APB**  **FRET Eff. (%)** | **Sequential APB**  **R2 Value** |
| --- | --- | --- | --- | --- | --- |
|  |  |  |  |  |  |
| **Cell 1** | 0.2041 | 8.52 | Bleached | 11.27 | 0.9683 |
|  |  | -0.63 | Unbleached |  |  |
|  |  |  |  |  |  |
| **Cell 2** | 0.2543 | 14.30 | Bleached | 14.13 | 0.9988 |
|  |  | -0.42 | Unbleached |  |  |
|  |  |  |  |  |  |
| **Cell 3** | 0.1689 | 6.74 | Bleached | 7.52 | 0.9874 |
|  |  | 0.91 | Unbleached |  |  |
|  |  |  |  |  |  |
| **Cell 4** | 0.2107 | 13.23 | Bleached | 13.91 | 0.9956 |
|  |  | -0.71 | Unbleached |  |  |
|  |  |  |  |  |  |
| **Cell 5*** | 0.1323 | 13.05 | Bleached | 16.13 | 0.9864 |
|  |  | 0.77 | Unbleached |  |  |
|  |  |  |  |  |  |

| **CFP-BAR1 +**  **BAR2-YFP**  **FRET Pair** | **Sensitized Emission**  **NFRET** | **Standard APB**  **FRET Eff. (%)** | **Standard APB**  **Cell Region** | **Sequential APB**  **FRET Eff. (%)** | **Sequential APB**  **R2 Value** |
| --- | --- | --- | --- | --- | --- |
|  |  |  |  |  |  |
| **Cell 1** | 0.3500 | 35.28 | Bleached | 37.02 | 0.9951 |
|  |  | 0.08 | Unbleached |  |  |
|  |  |  |  |  |  |
| **Cell 2** | 0.3172 | 31.99 | Bleached | 37.53 | 0.9926 |
|  |  | 1.41 | Unbleached |  |  |
|  |  |  |  |  |  |
| **Cell 3** | 0.2419 | 21.34 | Bleached | 24.54 | 0.9982 |
|  |  | -3.14 | Unbleached |  |  |
|  |  |  |  |  |  |
| **Cell 4** | 0.2111 | 29.91 | Bleached | 31.98 | 0.9972 |
|  |  | 1.91 | Unbleached |  |  |
|  |  |  |  |  |  |
| **Cell 5*** | 0.2546 | 28.09 | Bleached | 30.06 | 0.9979 |
|  |  | 3.02 | Unbleached |  |  |
|  |  |  |  |  |  |

| **CFP-BAR2 + YFP**  **FRET Pair** | **Sensitized Emission**  **NFRET** | **Standard APB**  **FRET Eff. (%)** | **Standard APB**  **Cell Region** | **Sequential APB**  **FRET Eff. (%)** | **Sequential APB**  **R2 Value** |
| --- | --- | --- | --- | --- | --- |
|  |  |  |  |  |  |
| **Cell 1** | 0.0997 | 0.10 | Bleached | -13.31 | 0.0061 |
|  |  | -4.04 | Unbleached |  |  |
|  |  |  |  |  |  |
| **Cell 2** | 0.0713 | 3.35 | Bleached | 4.06 | 0.8524 |
|  |  | -3.17 | Unbleached |  |  |
|  |  |  |  |  |  |
| **Cell 3** | 0.1090 | 4.47 | Bleached | 6.95 | 0.9507 |
|  |  | -0.66 | Unbleached |  |  |
|  |  |  |  |  |  |
| **Cell 4** | 0.0971 | 9.31 | Bleached | 12.06 | 0.9723 |
|  |  | 3.18 | Unbleached |  |  |
|  |  |  |  |  |  |
| **Cell 5** | 0.1136 | 7.87 | Bleached | 12.03 | 0.9615 |
|  |  | 4.37 | Unbleached |  |  |
|  |  |  |  |  |  |

| **CFP-BAR2 +**  **BAR1-YFP**  **FRET Pair** | **Sensitized Emission**  **NFRET** | **Standard APB**  **FRET Eff. (%)** | **Standard APB**  **Cell Region** | **Sequential APB**  **FRET Eff. (%)** | **Sequential APB**  **R2 Value** |
| --- | --- | --- | --- | --- | --- |
|  |  |  |  |  |  |
| **Cell 1** | 0.1224 | 5.57 | Bleached | 6.42 | 0.9799 |
|  |  | -1.68 | Unbleached |  |  |
|  |  |  |  |  |  |
| **Cell 2** | 0.1305 | 8.28 | Bleached | 9.32 | 0.9924 |
|  |  | -1.28 | Unbleached |  |  |
|  |  |  |  |  |  |
| **Cell 3** | 0.1173 | 4.29 | Bleached | 5.67 | 0.9342 |
|  |  | -3.46 | Unbleached |  |  |
|  |  |  |  |  |  |
| **Cell 4** | 0.1262 | 6.33 | Bleached | 7.41 | 0.9885 |
|  |  | -2.00 | Unbleached |  |  |
|  |  |  |  |  |  |
| **Cell 5** | 0.1017 | 9.76 | Bleached | 11.36 | 0.9914 |
|  |  | 1.05 | Unbleached |  |  |
|  |  |  |  |  |  |

| **CFP-BAR2 +**  **BAR2-YFP**  **FRET Pair** | **Sensitized Emission**  **NFRET** | **Standard APB**  **FRET Eff. (%)** | **Standard APB**  **Cell Region** | **Sequential APB**  **FRET Eff. (%)** | **Sequential APB**  **R2 Value** |
| --- | --- | --- | --- | --- | --- |
|  |  |  |  |  |  |
| **Cell 1** | 0.2465 | 22.76 | Bleached | 25.48 | 0.9978 |
|  |  | -5.62 | Unbleached |  |  |
|  |  |  |  |  |  |
| **Cell 2** | 0.2585 | 20.42 | Bleached | 21.56 | 0.9843 |
|  |  | -1.43 | Unbleached |  |  |
|  |  |  |  |  |  |
| **Cell 3** | 0.2630 | 20.32 | Bleached | 22.58 | 0.9969 |
|  |  | 0.09 | Unbleached |  |  |
|  |  |  |  |  |  |
| **Cell 4** | 0.2847 | 28.87 | Bleached | 30.46 | 0.9979 |
|  |  | -0.67 | Unbleached |  |  |
|  |  |  |  |  |  |
| **Cell 5** | 0.2526 | 22.34 | Bleached | 24.30 | 0.9954 |
|  |  | 0.32 | Unbleached |  |  |
|  |  |  |  |  |  |
| **Cell 6** | 0.2318 | 19.99 | Bleached | 23.89 | 0.9953 |
|  |  | -0.55 | Unbleached |  |  |
|  |  |  |  |  |  |
| **Cell 7*** | 0.2897 | 34.11 | Bleached | 36.04 | 0.9986 |
|  |  | 2.29 | Unbleached |  |  |
|  |  |  |  |  |  |
