# Supplementary material for: APPL Proteins FRET at the BAR: Direct Observation of APPL1 and APPL2 BAR Domain-Mediated Interactions on Cell Membranes Using FRET Microscopy
Source: PLoS One. 2010 Aug 30;5(8):e12471. doi: 10.1371/journal.pone.0012471 (PMC2930004; doi:10.1371/journal.pone.0012471)
Supplement: Table S2 — Summary of average FRET values, standard deviations, and p-values for the nine FRET pairs. (0.14 MB DOC) [file pone.0012471.s003.doc]

**Table S2. Summary of average FRET values, standard deviations, and p-values for the nine FRET pairs.**

| **FRET Pair** | **Sensitized Emission**  **NFRET** | **Standard APB**  **FRET Eff. (%)** | **Standard APB**  **Cell Region** | **Sequential APB**  **FRET Eff. (%)** | **Sequential APB**  **R2 Value** |
| --- | --- | --- | --- | --- | --- |
|  |  |  |  |  |  |
|  |  |  |  |  |  |
| **CFP + YFP:** | 0.0213 +/- 0.0047 | 4.06 +/- 1.57 | Bleached | 5.12 +/- 0.82 | 0.6890 +/-0.2094 |
|  |  | -3.99 +/- 2.26 | Unbleached |  |  |
|  |  |  |  |  |  |
| **CFP + BAR1-YFP:** | 0.0210 +/- 0.0109 | -1.59 +/- 7.90 | Bleached | -2.00 +/- 11.10 | 0.5382 +/- 0.3185 |
|  |  | -4.74 +/- 2.19 | Unbleached |  |  |
|  |  |  |  |  |  |
| **CFP + BAR2-YFP:** | 0.0425 +/- 0.0198 | -18.51 +/- 9.46 | Bleached | -21.12 +/- 9.85 | 0.8208 +/- 0.0966 |
|  |  | -4.63 +/- 2.23 | Unbleached |  |  |
|  |  |  |  |  |  |

| **FRET Pair** | **Sensitized Emission**  **NFRET** | **Standard APB**  **FRET Eff. (%)** | **Standard APB**  **Cell Region** | **Sequential APB**  **FRET Eff. (%)** | **Sequential APB**  **R2 Value** |
| --- | --- | --- | --- | --- | --- |
|  |  |  |  |  |  |
|  |  |  |  |  |  |
| **CFP-BAR1 + YFP:** | 0.0299 +/- 0.0067 | 5.12 +/- 1.40 | Bleached | 5.88 +/- 1.75 | 0.9332 +/- 0.0738 |
|  |  | -0.22 +/- 1.95 | Unbleached |  |  |
|  |  |  |  |  |  |
| **CFP-BAR1 + BAR1-YFP:** | 0.1941 +/- 0.0460 | 11.17 +/- 3.32 | Bleached | 12.59 +/- 3.32 | 0.9873 +/- 0.0119 |
|  |  | -0.02 +/- 0.79 | Unbleached |  |  |
| **p values for CFP-BAR1 + BAR1-YFP: (PE=Pooled, Equal; SU=Satterthwaite, Unequal):** | | | | |  |
| vs. CFP + YFP | 0.0010 (SU) | 0.0025 (PE) |  | 0.0061 (SU) |  |
| vs. CFP + BAR1-YFP | 0.0008 (SU) | 0.0104 (PE) |  | 0.0398 (SU) |  |
| vs. CFP-BAR1 + YFP | 0.0001 (PE) | 0.0056 (PE) |  | 0.0040 (PE) |  |
|  |  |  |  |  |  |
| **CFP-BAR1 + BAR2-YFP:** | 0.2750 +/- 0.0570 | 29.32 +/- 5.20 | Bleached | 32.23 +/- 5.36 | 0.9962 +/- 0.0023 |
|  |  | 0.66 +/- 2.37 | Unbleached |  |  |
| **p values for CFP-BAR1 + BAR2-YFP: (PE=Pooled, Equal; SU=Satterthwaite, Unequal):** | | | | |  |
| vs. CFP + YFP | 0.0005 (SU) | 0.0002 (SU) |  | 0.0003 (SU) |  |
| vs. CFP + BAR2-YFP | <0.0001 (PE) | <0.0001 (PE) |  | <0.0001 (PE) |  |
| vs. CFP-BAR1 + YFP | 0.0006 (SU) | 0.0003 (SU) |  | <0.0001 (PE) |  |
|  |  |  |  |  |  |

| **FRET Pair** | **Sensitized Emission**  **NFRET** | **Standard APB**  **FRET Eff. (%)** | **Standard APB**  **Cell Region** | **Sequential APB**  **FRET Eff. (%)** | **Sequential APB**  **R2 Value** |
| --- | --- | --- | --- | --- | --- |
|  |  |  |  |  |  |
|  |  |  |  |  |  |
| **CFP-BAR2 + YFP:** | 0.0981 +/- 0.0164 | 5.02 +/- 3.67 | Bleached | 4.36 +/- 10.45 | 0.7486 +/- 0.4178 |
|  |  | -0.06 +/- 3.74 | Unbleached |  |  |
|  |  |  |  |  |  |
| **CFP-BAR2 + BAR1-YFP:** | 0.1196 +/- 0.0111 | 6.85 +/- 2.18 | Bleached | 8.04 +/- 2.31 | 0.9773 +/- 0.0246 |
|  |  | -1.47 +/- 1.63 | Unbleached |  |  |
| **p values for CFP-BAR2 + BAR1-YFP: (PE=Pooled, Equal; SU=Satterthwaite, Unequal):** | | | | |  |
| vs. CFP + YFP | <0.0001 (SU) | 0.0487 (PE) |  | 0.0287 (PE) |  |
| vs. CFP + BAR1-YFP | <0.0001 (PE) | 0.0740 (SU) |  | 0.1132 (SU) |  |
| vs. CFP-BAR2 + YFP | 0.0419 (PE) | 0.3665 (PE) |  | 0.4816 (SU) |  |
|  |  |  |  |  |  |
| **CFP-BAR2 + BAR2-YFP:** | 0.2610 +/- 0.0205 | 24.12 +/- 5.36 | Bleached | 26.33 +/- 5.15 | 0.9952 +/- 0.0050 |
|  |  | -0.80 +/- 2.43 | Unbleached |  |  |
| **p values for CFP-BAR2 + BAR2-YFP: (PE=Pooled, Equal; SU=Satterthwaite, Unequal):** | | | | |  |
| vs. CFP + YFP | <0.0001 (SU) | <0.0001 (SU) |  | <0.0001 (SU) |  |
| vs. CFP + BAR2-YFP | <0.0001 (PE) | <0.0001 (PE) |  | <0.0001 (PE) |  |
| vs. CFP-BAR2 + YFP | <0.0001 (PE) | <0.0001 (PE) |  | 0.0007 (PE) |  |
|  |  |  |  |  |  |
